# Regularly irregular tachycardia and QRS alternans? Think twice!

**DOI:** 10.1093/ehjcr/ytae150

**Published:** 2024-03-26

**Authors:** João Grade Santos, Sofia Almeida, Luís Brandão

**Affiliations:** Cardiology Registrar, Hospital Garcia de Orta, Avenida Torrado da Silva, 2805-267 Almada, Portugal; Cardiology Registrar, Hospital Garcia de Orta, Avenida Torrado da Silva, 2805-267 Almada, Portugal; Cardiology Registrar, Hospital Garcia de Orta, Avenida Torrado da Silva, 2805-267 Almada, Portugal

## ECG Challenge

A 67-year-old male, with no relevant priors, was referred to Electrophysiology (EP) evaluation after failing multiple antiarrhythmic medical therapy (beta-blockers, propafenone, and sotalol) for highly symptomatic paroxysms of tachyarrhythmia.

The patient showed two different 12-lead electrocardiograms (ECG) shown below (*Panels A* and *B*).

Additional Holter monitoring is provided in Supplementary material online, *Figure S1*.

**Question 1:** Panel A demonstrates sinus rhythm with a brief period of regularly irregular QRS alternans tachycardia with a P wave preceding only every other QRS complex (as further depicted in the Supplementary material online, *Figure S1*).

Panel B demonstrates a regular rhythm with narrow QRS complexes with inferior axis P waves visualized after the QRS at the initial part of the ST segment.

Which of the following could not explain the observed tachyarrhythmia?

- Atrial Bigeminy with aberrancy.
- Ventricular Bigeminy.
- Atrioventricular nodal re-entrant tachycardia (AVNRT) with 2:1 retrograde block.
- Dual atrioventricular nodal non-reentrant tachycardia (DAVNNT).
- Ortodromic atrioventricular re-entrant tachycardia (AVRT).

**Correct answer: e)**

**Explanation:** In the differential diagnosis of the narrow-complex tachycardia with a P:R ratio of 1:2 and QRS alternans between narrow and wide complex one must consider either ventricular bigeminy, atrial bigeminy with the latter *P*-wave entailed in the preceding T wave and some degree of aberrancy, AVNRT with 2:1 retrograde block and DAVNNT also known as ‘double fire’ tachycardia (as depicted in Supplementary material online, *Figure S2*).^1^

An AVRT could not produce a narrow complex tachycardia with a P:R ratio of 1:2 as both atria and ventricle are part of the arrythmia circuit.

**Question 2:** Given the most likely diagnosis, what would be the expected findings in the EP study?

- An accessory pathway.
- Dual nodal physiology.
- An atrial focus.
- A ventricular focus.
- A macro-reentrant circuit.

**Correct answer: b)**

**Explanation:** Panel A could be interpreted as nodal conduction through a fast pathway followed by simultaneous conduction through a slow and fast pathway and Panel B uniquely conduction through a slow pathway.

As such, the expected finding would be dual nodal physiology. In this patient, an EP study was conducted (see Supplementary material online, *Figures S3–S5*) and dual nodal physiology was identified with the presence of AH jump and induction of AVNRT. At the end of an atrial pacing drive, there was a period in which each atrial beat produced two slightly different QRS complexes, thus making the case for DAVNNT.

**Question 3:** Given the most likely EP study findings, what would be the most appropriate management?

- Ablation of the focal arrhythmia.
- Ablation of the accessory pathway.
- Ablation of the slow pathway.
- Ablation of the macro-reentrant circuit.
- Antiarrhythmic drug therapy.

**Correct answer: c)**

**Explanation:** Dual atrioventricular nodal non-reentrant tachycardia occurs in patients with dual AV physiology in which each *P* wave conducts anterogradely both down the fast and slow pathways producing two distinct QRS complexes. Although this is a rare entity, it can result in tachycardia-induced cardiomyopathy and is easily treated by catheter ablation of the slow pathway.^2^ In this patient, a slow pathway ablation was performed with resolution of symptoms and electrocardiographic findings.

**Figure:**
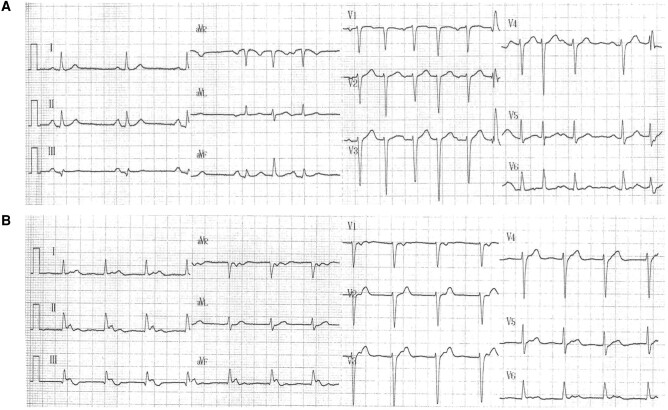


**Consent:** Direct written consent was obtained by the patient for this manuscript.

**Funding:** No funding was involved in the development of this manuscript.

## Supplementary Material

ytae150_Supplementary_Data

## Data Availability

The data that support the findings of this study are available from the authors upon request.
