# Supplementary material for: Regularly irregular tachycardia and QRS alternans? Think twice!
Source: Eur Heart J Case Rep. 2024 Mar 26;8(4):ytae150. doi: 10.1093/ehjcr/ytae150 (PMC10986392; doi:10.1093/ehjcr/ytae150)
Supplement: ytae150_Supplementary_Data [file ytae150_supplementary_data.pdf]

Supplementary Figures

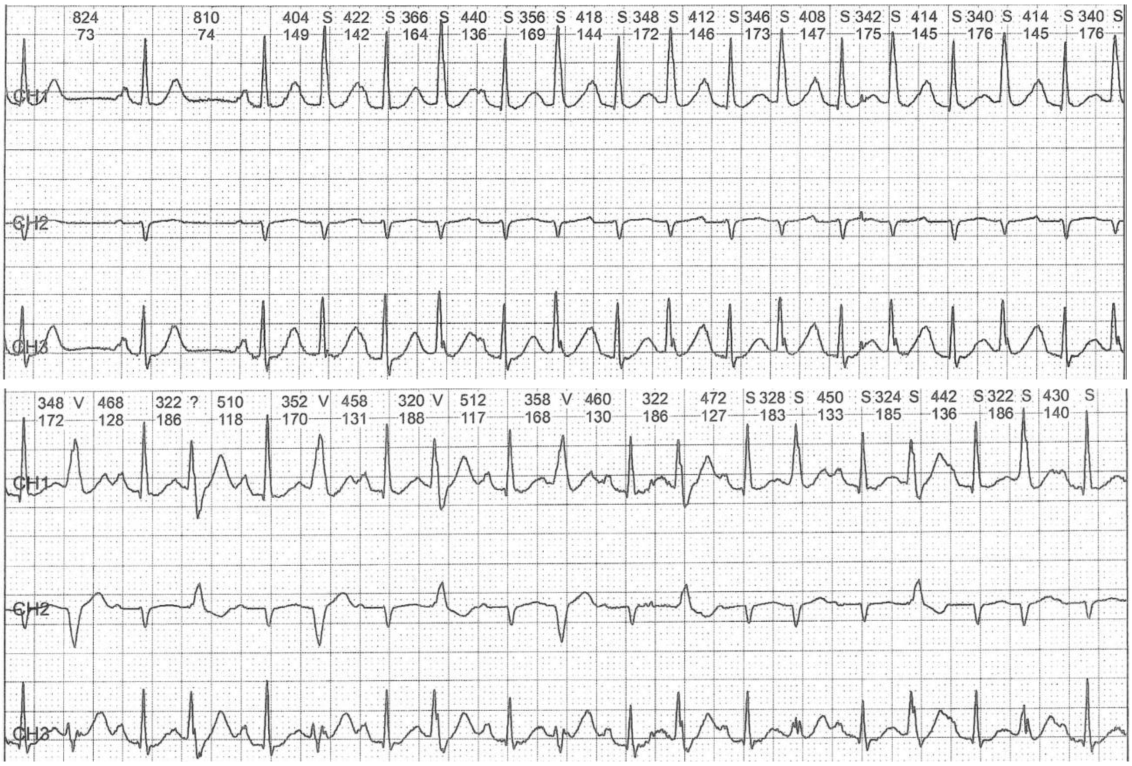

Supplementary Figure 1 An Holter monitoring showing initial sinus rhythm which initiates a regularly irregular tachycardia with QRS alternans (between narrow complex and slightly aberrant complexes) with a P wave entailed in every other T wave giving an apparent P:R ratio of 1:2.

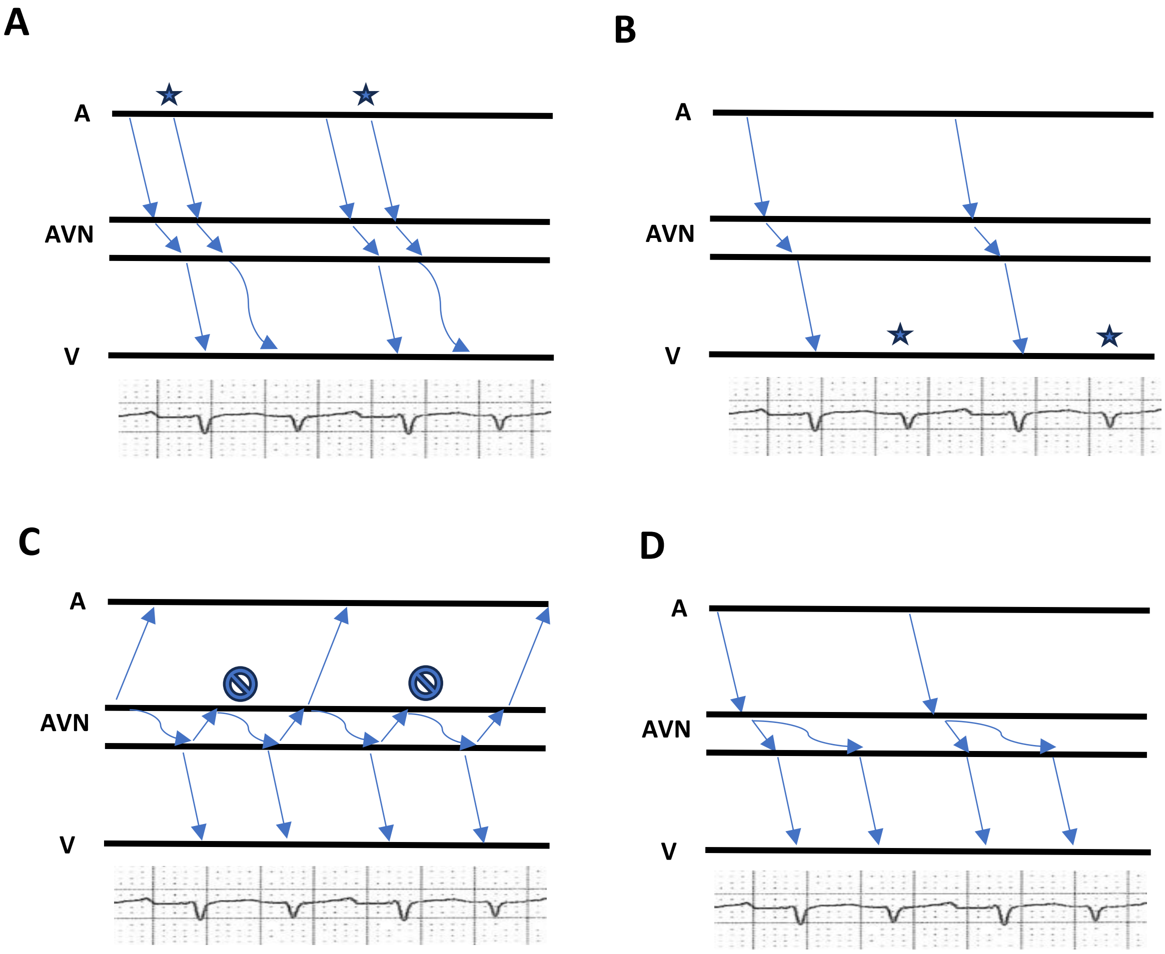

Supplementary Figure 2 A diagrammatic explanation for the differential diagnosis of a narrow-complex tachycardia with a P:R ratio of 1:2. Panel A represents Atrial Bigeminy with aberrancy, panel B Ventricular Bigeminy, panel C AVNRT with 2:1 retrograde block and panel D DAVNNT.

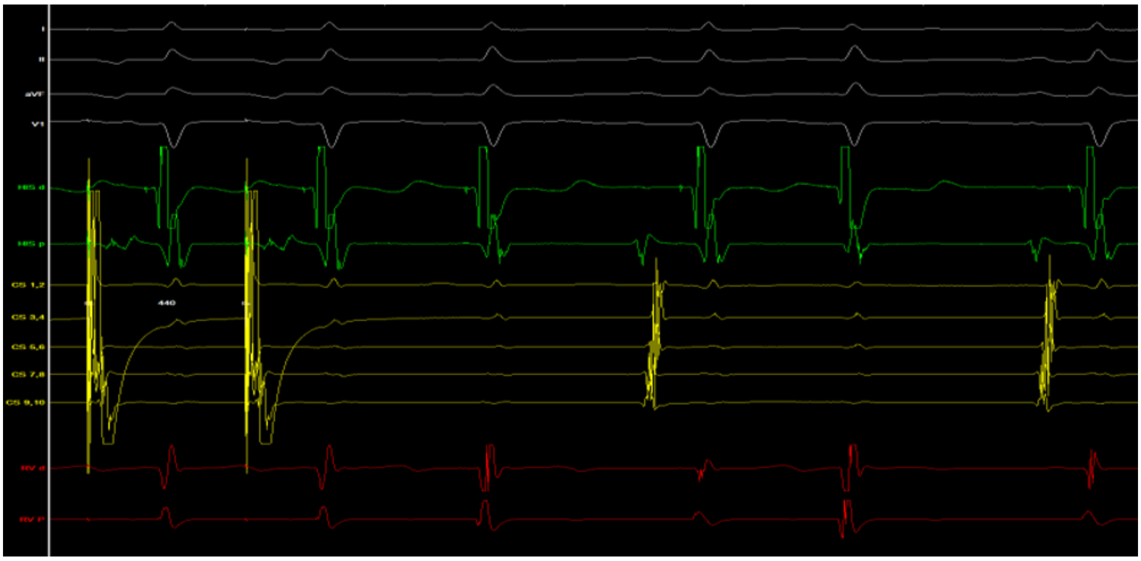

Supplementary Figure 3 Surface and intracardiac electrocardiograms at the end of 440 ms atrial pacing drive train. The last atrial paced is followed two His and ventricular electrograms. The first intrinsic sinus beat is also followed by two His and ventricular electrograms with slightly different QRS morphology in the surface ECG (with the disappearance of the initial R wave in V1 and higher voltage R wave in aVF in the second produced QRS).

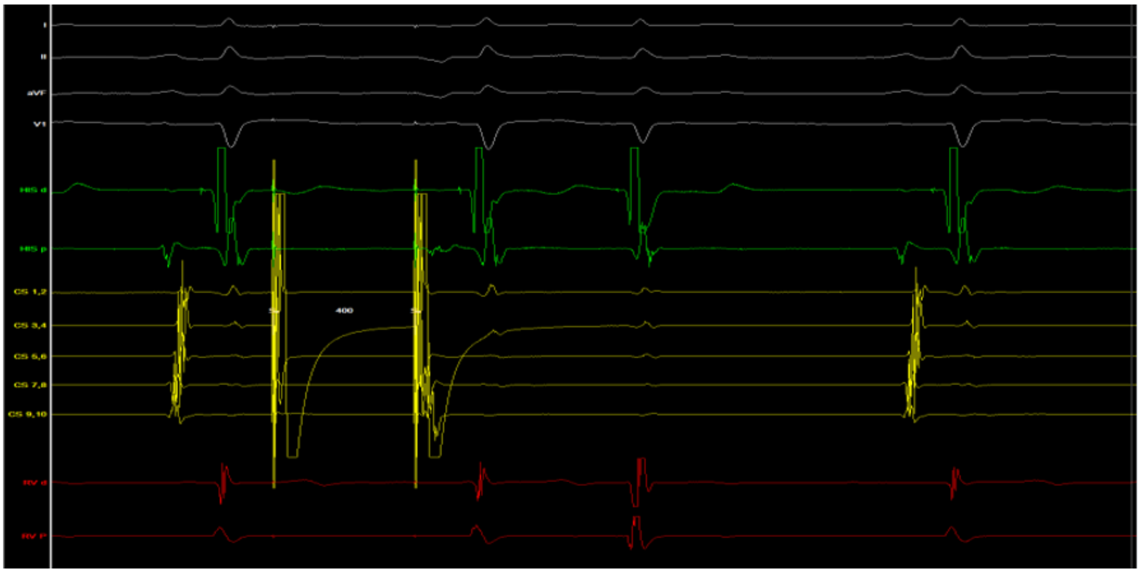

Supplementary Figure 4 Surface and intracardiac electrocardiograms after sensed coupled atrial paced beats. The second atrial paced beat is followed by two His and ventricular electrograms. This constant and reproducible finding, as well as the stable time interval between the atrial paced beat and the second ventricular electrogram argues against it being a junctional beat. The non-inducibility of this finding after slow pathway ablation adds further proof.

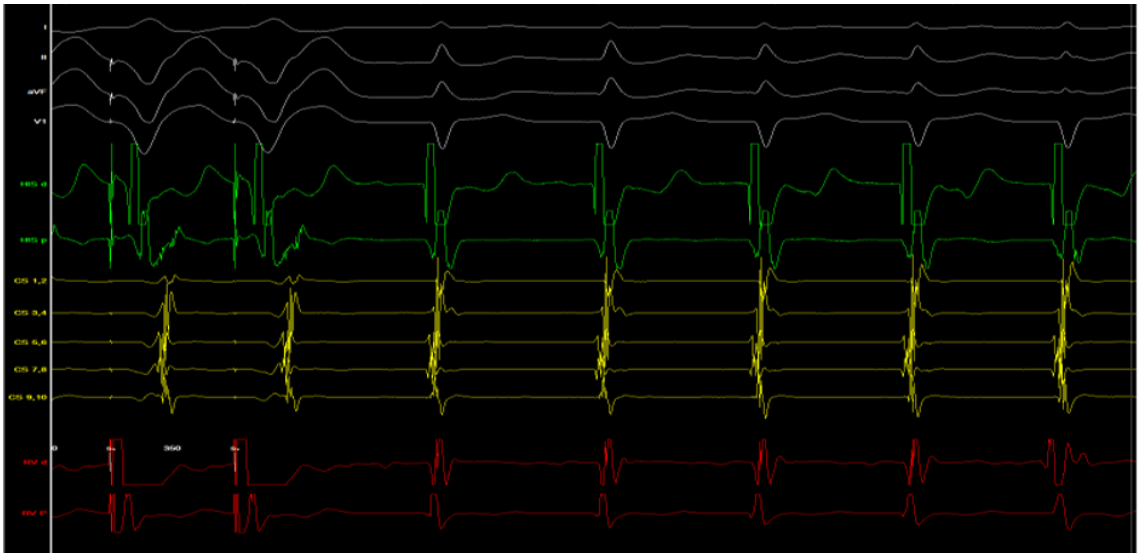

Supplementary Figure 5 Surface and intracardiac electrocardiograms at the end of 350ms ventricular pacing drive train inducing a regular narrow complex tachycardia with VA interval of 0 which, with proven dual AV node physiology, is suggestive of the AVRNT.
